# Development and Testing of a Decision Making Based Method to Adjust Automatically the Harrowing Intensity

**DOI:** 10.3390/s130506254

**Published:** 2013-05-13

**Authors:** Victor Rueda-Ayala, Martin Weis, Martina Keller, Dionisio Andújar, Roland Gerhards

**Affiliations:** Department of Weed Science (360b), University of Hohenheim, 70599 Stuttgart, Germany; E-Mails: martin.weis@uni-hohenheim.de (M.W.); martina.keller@agroscope.admin.ch (M.K.); andujar@uni-hohenheim.de (D.A.); roland.gerhards@uni-hohenheim.de (R.G.)

**Keywords:** site-specific harrowing, selectivity, crop-weed-soil variability, crop-weed-soil sensors, fuzzy logic, precision weed control

## Abstract

Harrowing is often used to reduce weed competition, generally using a constant intensity across a whole field. The efficacy of weed harrowing in wheat and barley can be optimized, if site-specific conditions of soil, weed infestation and crop growth stage are taken into account. This study aimed to develop and test an algorithm to automatically adjust the harrowing intensity by varying the tine angle and number of passes. The field variability of crop leaf cover, weed density and soil density was acquired with geo-referenced sensors to investigate the harrowing selectivity and crop recovery. Crop leaf cover and weed density were assessed using bispectral cameras through differential images analysis. The draught force of the soil opposite to the direction of travel was measured with electronic load cell sensor connected to a rigid tine mounted in front of the harrow. Optimal harrowing intensity levels were derived in previously implemented experiments, based on the weed control efficacy and yield gain. The assessments of crop leaf cover, weed density and soil density were combined via rules with the aforementioned optimal intensities, in a linguistic fuzzy inference system (LFIS). The system was evaluated in two field experiments that compared constant intensities with variable intensities inferred by the system. A higher weed density reduction could be achieved when the harrowing intensity was not kept constant along the cultivated plot. Varying the intensity tended to reduce the crop leaf cover, though slightly improving crop yield. A real-time intensity adjustment with this system is achievable, if the cameras are attached in the front and at the rear or sides of the harrow.

## 1. Introduction

Mechanical weed control provides a good alternative to reduce weed pressure, in both organic and conventional farming. The heterogeneous spatial and temporal distribution of weed populations causes underestimation of potential yield loss in areas with high weed densities or overestimation in areas with low or no weed densities [1]. This has opened an opportunity to develop strategies for site-specific mechanical weed control and thereby to reduce environmental and economic costs associated with weed control treatments. Machine vision, global positioning systems (GPS), variable rate application systems and robotics are providing technological tools to allow autonomous control of weeding implements to become feasible [2]. Moreover, site-specific weed management strategies have been investigated under variable field conditions [3]. Advances on GPS-guidance of intra-row hoes or automatic control of finger weeders provide promising prospects to achieve site-specific mechanical weed control [4–6].

Harrowing with a flexible tine weeder is commonly used to reduce weed competition in cereals and legumes. Generally, a constant harrowing intensity is applied across the whole field, regardless of variations in weed distribution and soil structure. Keeping a constant harrowing intensity for the whole field may result in crop damage due to an aggressive treatment in areas with low weed infestations, young and small weeds or light soil density. Similarly, a gentler intensity may generate yield losses due to insufficient weed control in high weed infestation patches. Weed harrowing controls weeds by uprooting or covering weed seedlings with soil, but the crop may also get covered with soil or torn into pieces [7]. The draught force opposite to the direction of travel is the specific resistance that the harrow tines should overcome to cultivate the soil [8]. Areas with loose soil conditions would be more aggressively harrowed than areas with denser soil, and these variations in soil conditions might lead to uneven weed control [5]. To increase the harrowing efficacy and balance the trade-off between crop damage and weed control, the applied intensity should be adapted to the variability of soil, weeds and crop within a field.

Harrowing intensity refers to the cultivation aggressiveness of the tines penetrating into the soil surface. Higher intensity levels are achieved by decreasing the tine angle relative to a perpendicular axis to the field surface, increasing the depth of the implement, increasing driving speed or through various consecutive passes on the same day of cultivation [6,9,10]. The crop-weed selectivity of harrowing and crop recovery have been studied as key relationships to determine the optimal harrowing intensity [11–13]. Implement settings have not been investigated, thus they were a topic of this study. Selectivity refers to the ratio between weed control percentage and the percentage of soil covering the crop, immediately after harrowing and excluding weed recovery or new weed emergence [14]. Crop recovery refers to the ability of the crop to tolerate burial in soil and to avoid yield losses as a result of harrowing. Selectivity and crop recovery from harrowing have been determined with objective assessment and analysis methods [10,11]. However, the non-uniformity of weed occurrence and soil conditions has been left aside. Conversely, in other studies weed spatial variability and soil density have been used in systems to automatically control the harrowing intensity, but selectivity and crop recovery were not taken into account [5,6]. Therefore, a system gathering all these aspects is still required.

The aim of this study was to develop and test a decision making based method to automatically adjust the harrowing intensity by varying the tine angle and number of passes. For this purpose, assessment of the crop-weed-soil variability was a requisite, as well as the intensity optimization through analysis of selectivity and crop recovery. Results of previous studies were used to determine optimal intensity levels, which became the output of the decision system [15,16]. These optimal intensity levels generated yield gain as a result of reduction of weed competition, in some cases even comparable with the effects of herbicide application [16]. It was assumed that these optimal intensities could be applicable in other fields with variable conditions. Since selectivity and crop recovery are relationships established after harrowing operations, they could not be included into the decision making method to adjust the intensity. Instead, the assessed variables (crop) leaf cover and weed density were used as inputs. In early post-emergence harrowing, leaf cover refers to crop plants because weed cover is insignificant [10]. In addition, the draught force of the soil (soil density) was assessed and included as another input into this decision making method. Hypotheses in this study included the following. (i) Leaf cover, weed density and soil density and the applied harrowing intensities in a previous experimental phase can be used to create a decision making based method for the automatic control of the harrowing intensity. Therefore, simple rules were formulated in a linguistic fuzzy inference system (LFIS) to combine input from bi-spectral cameras estimating crop leaf cover and weed density and a soil sensor; (ii) Harrowing according to the assessed variability is achievable, and site-specific harrowing effectively diminishes crop damage due to harrowing, while maintaining high levels of weed control and increasing crop yield. For this, application maps were created using the aforementioned decision making based method and applied in two field experiments. With these experiments it was intended to determine effects of keeping constant harrowing intensities along plots compared with applying sensor-based variable intensities. Fuzzy logic allows to implement human reasoning in computing technology through an interface between symbolic and numerical spaces [17]. Therefore, the method proposed here contributed a tool to deal with the contextual definition of harrowing intensity, which may vary from light to high levels depending on the implement settings.

## 2. Materials and Methods

### 2.1. Data Source

A decision system for automatic harrowing was developed based on results of previously carried out experiments in winter and spring cereals, barley (*Hordeum vulgare* L.) and wheat (*Triticum aestivum* L.). With these experiments it was possible to determine the influence of crop growth stage and harrowing intensity on selectivity and crop yield. The experimental period ranged from 2007 to 2009, at different sites with varying soil conditions and weed densities (Table 1). Further details on experiments 1 to 4 are given in Rueda-Ayala and Gerhards [15] and Rueda-Ayala *et al.* [16]. Experiments 5 and 6 contain previously unpublished data [18], but the variable assessment and analysis procedure were the same as for experiments 1 to 4.

The experimental sites were located at three research stations of the University of Hohenheim: Heidfeldhof (48°43′N, 9°12′E) and Meiereihof (48°43′N, 9°15′E), near Stuttgart, and Ihinger Hof (48°45′ N, 8°56′ E), near Renningen. In Rueda-Ayala *et al.* [16], only experiments conducted in Germany were used so as to keep homogeneous characteristics of harrowing implements and weed- and soil assessments. Harrowing was performed with a 6-m-wide flexible-tine harrow (Hatzenbichler Austrian Agrotechnik). Different tine angles (see below) were combined with driving speeds from 8 to 10 km h^−1^ and up to two passes to create increasingly aggressive intensities, including one untreated control. Decisions about speed, angles and settings were based on visual assessments on the whole field, at the day of harrowing such that one pass covered between 25% and 30% of the crop with soil. The analysis procedures for studying selectivity provided in Rasmussen *et al.* [11] and Rasmussen *et al.* [12] were used. The yield responses to weed control by harrowing was analyzed as in Rueda-Ayala *et al.* [16].

The approach for sensor-based mechanical weed control outlined in Weis *et al.* [1] was used. Crop leaf cover and weed density were assessed using bispectral cameras. The bispectral camera has two channels, infrared (IR) and visual (VIS), which take two images at the same time in the near-infrared (770–1,150 nm) and the red (610–670 nm) spectra. The images are subtracted, *i.e.*, IR–VIS, resulting in a differential image with strong contrast between green plants and soil, mulch and stones. Nearly one image per second was acquired to measure crop leaf cover and weed density. For this, two bispectral cameras separated 2.3 m from left to right were mounted on a vehicle driven at 5 km h^−1^. The weed density was also manually counted to verify the results of the digital image analysis. Automated assessment of weed density was carried out in all experiments.

**Figure 1.**
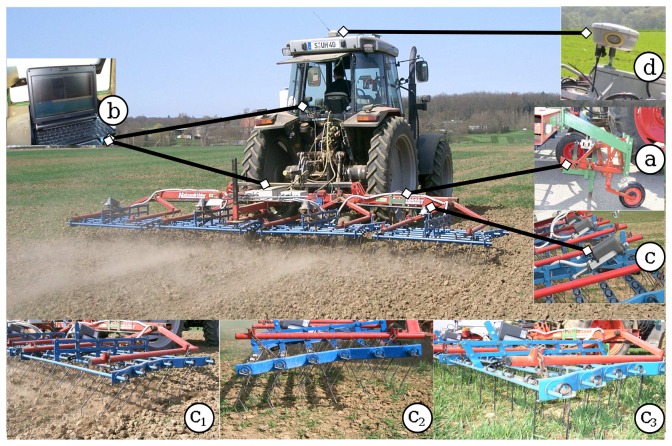
Prototype of the automatically controlled flexible-tine harrow, adapted from Rueda-Ayala *et al.* [19]. (**a**) Soil sensor; (**b**) computing unit; (**c**) motor; (c_1_) light intensity; (c_2_) strong intensity; (c_3_) strongest intensity; (**d**) RTK-DGPS.

Figure 1 shows the developed prototype for automatic harrowing. The soil density was assessed with an electronic load cell sensor (Tedea-Huntleigh's model 615 S-type, Tedea-Huntleigh GmbH, Darmstadt, Germany). A rigid tine connected to the sensor was mounted on the harrow to penetrate the soil to a depth of ±3 cm (Figure 1(a)). The draught force offered by the soil to the rigid tine is almost equal at this shallow depth, because the produced soil disturbance (e.g., by the harrow tines) is similar [8]. The applied force was measured with the aid of a spring with a known spring constant and a strain gauge that measures displacement and outputs it as voltage. Based on Hooke's law, the displacement measured by the strain gauge can be attributed to specific force applied to the end of the tine. Approximately 30 measurement points per second were acquired at a driving speed of 8 km h^−1^. Horizontal movements of the rigid tine were captured and the variation of voltages were calibrated to different force levels measured in Newton (data not shown). Soil density could not be measured in all experiments due to technical difficulties; however, a good differentiation in soil between winter and spring cereals was identified. Soil density data and application maps (see Section 2.3) were sent to the computing unit (Figure 1(b)). This controlled the actuator (Figure 1(c)) to vary the harrowing intensity from the lightest to the strongest level (Figure 1(c_1_–c_3_)), according to the measured variability. A precise positioning system RTK-DGPS Trimble ® 5800 Limited GPS System 2001 was mounted on the system (Figure 1(d)), which enabled elaboration of crop leaf cover and weed density maps. Increasingly aggressive treatments were made by decreasing the tine angle relative to a perpendicular axis to the field surface. These harrowing intensity levels were: untreated or none (72°), lightest (61°), light (41°), strong (28°), and strongest (4°).

### 2.2. Decision Making Based Method for Automatic Harrowing

**Figure 2.**
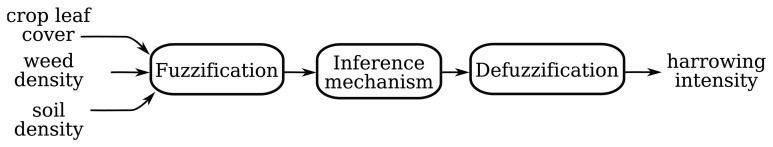
Inner structure of the fuzzy inference system decision making based method to control the harrowing intensity. The inference and the involved processes of fuzzification and defuzzification are based on if-then rules. Adapted from Xia *et al.* [20].

A linguistic fuzzy logic decision making based method was developed to control the adjustment of the harrowing intensity according to three input variables: crop leaf cover, weed density and soil density. The harrowing intensities from the experiments in 2007–2009, which achieved high selectivity, high crop yield gains or negligible crop yield reductions (Table 2), were assigned as the optimal output intensities, as described below. The mathematical principle to construct this decision making based method was a fuzzy rule-based inference system (Figure 2). This system has three main components: fuzzification interface (fuzzy sets), inference mechanism based on if-then rules, and defuzzification interface [20]. A broader description of fuzzy logic has been outlined by several authors [17,21–23].

**Figure 3.**
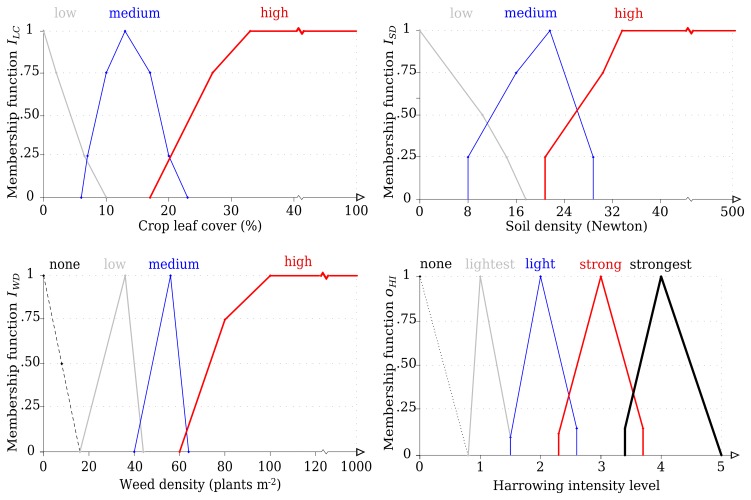
Membership functions of the input variables crop leaf cover (*I_LC_*), weed density (*I_WD_*), soil density (*I_SD_*) and of the output variable harrowing intensity (*O_HI_*).

The fuzzification interface received the assessed numeric inputs: crop leaf cover, weed density and soil density. These values were translated into “fuzzy sets” characterizing the linguistic variables crop leaf cover (*I_LC_*), weed density (*I_WD_*), soil density (*I_SD_*). The optimal harrowing intensities defined (none, lightest, light, strong, strongest) and tested in previous experiments were also fuzzified into the fuzzy set harrowing intensity (*O_HI_*). All fuzzy sets were characterized through membership functions (*MF*) with a continuum degree of membership. Three *MF* were determined for *I_LC_* and *I_SD_*, and four for *I_WD_* (Figure 3). The universes of discourse for the fuzzy sets were determined as explained below. Crop leaf cover measured at BBCH 12–14 depicted the “low” MF of *I_LC_*; older growth stages, such as BBCH 15–21 and BBCH 22–31, characterized the medium and high levels, respectively. At a crop leaf cover lower than 2%, the crop would not resist being harrowed, but with more than 40% leaf cover, the crop could withstand an aggressive harrowing intensity. A high weed competition was assumed with 100 weeds m^−2^ or more, at which harrowing must be applied with maximum intensity level. No weed competition was assigned at a density below 15 weeds m^−2^. For *I_SD_*, higher membership degrees than 30 N indicated a highly dense soil, in which weed harrowing would not be favorable due to poor soil workability [19].

*MF* for *O_HI_* were defined using the same intensity levels from the data source (Table 2), as “none” (*i.e.*, untreated), “light”, “lightest”, “strong” and “strongest”. Intensities that achieved high weed control with low crop soil cover and yield gain or yield loss due to harrowing not higher than 3% were used. All levels of the fuzzy sets *I_LC_*, *I_WD_* and *I_SD_* were inserted into the inference mechanism. This mechanism applied a predefined set of rules to infer the output *O_HI_*, which is the fuzzy output with a degree of matching linguistic quantity. These rules basically consist of two parts: an IF “antecedent proposition” and THEN “consequent proposition” [24,25]. Thirty six rules (Table 3) were created using Boolean relations [26,27]. For instance, a rule is: IF *I_LC_* IS low AND *I_SD_* IS low AND *I_WD_* IS none THEN *O_HI_* IS none. Thus, the most influential input variable to infer a harrowing intensity was the weed density. Even if *I_WD_* is ‘none’, a gentle intensity might benefit the crop at ‘medium’ or ‘high’ soil densities (*I_SD_*) through soil loosening and reduction of evapotranspiration [30]. All rules in Table 3 were of general use because they were created using experimental data and objective variable assessment. The fuzzy output went through the defuzzification interface to be translated into numeric values, to enable its use in engineering applications [23,28]. The defuzzication method was center of gravity (CoG), which calculates the centroid from the integrated membership function [29].

### 2.3. Experimental Application of the System

To test the fuzzy inference system, two harrowing experiments (A and B) in winter wheat were conducted. The experiments were located at the Ihinger Hof research station, and the crop sown in autumn 2009. A split-strip plot design with four replication blocks was used in both trials. The harrowing treatments were applied to the whole length (80 m) of the main plots. The soil was rolled in strip-plots by one, two or three passes a few weeks after sowing. Each strip was 24 m long separated by a 4 m buffer-border to facilitate the rolling operations. The aim was to artificially generate three soil density levels along each experimental plot, thus enabling harrowing according to three known soil densities. The harrowing intensity levels none, lightest, light, strong and strongest were applied in twelve subplots, 6 m × 6 m within the main plot, as explained below.

In experiment A, the harrowing intensity levels none, light, strong and strongest were compared with a “fuzzy inferred variable intensity”, resulting in five treatments in total. Each intensity level was kept constant along the main plot, while the variable treatment consisted in varying those intensity levels in each subplot, within the main plot and based on the measured variability. Crop leaf cover *I_LC_*, weed density *I_WD_* and soil density *I_SD_* were assessed about two weeks before harrowing (not shown), as mentioned in Section 2.1. Technical difficulties did not allow to proceed with harrowing operations during the time lag after data assessment. Temperatures and rainfall from March 26th to April 9th, 2010 were on average lower than 4 ° C and 16 mm day^−1^, respectively. Thus, it was assumed that no significant plant growth or weed development existed.

**Figure 4.**
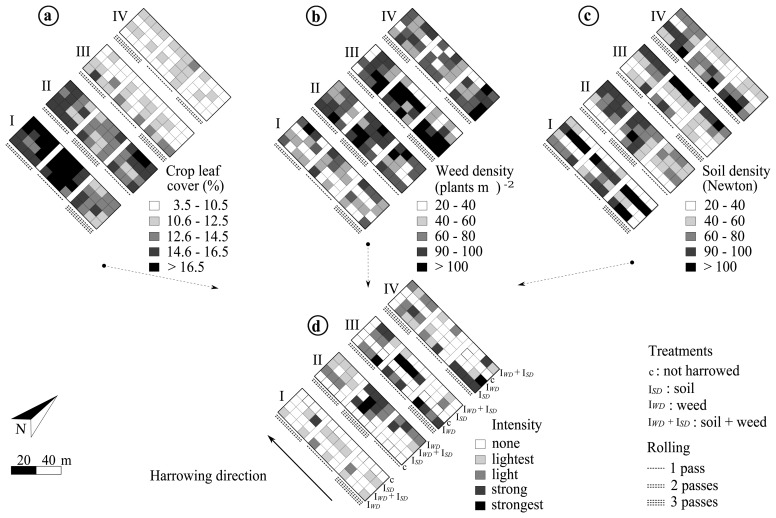
Experimental setup to the linguistic fuzzy inference system (LFIS) application (Experiment *B*). Inputs were obtained for crop leaf cover *I_LC_*, weed density *I*_WD_ and soil density *I_SD_* assessed before harrowing to infer the output harrowing intensity and create the application map.

In experiment B, three variable intensity treatments were compared with an untreated control (*c*). The harrowing intensity levels were determined based on three input sources: soil density only (*I_SD_*), weed density only (*I_WD_*), or a combination of both, soil and weed densities (*I_WD_* + *I_SD_*). Crop leaf cover (*I_LC_*) was included in all three possibilities as a reference of the crop growth stage. *I_LC_*, *I_WD_* and *I_SD_* were assessed before harrowing as described in Section 2.1 (Figure 4(a–c)). Six to eight images were acquired per subplot (6 m) to measure *I_LC_* and *I_WD_*, and nearly 75 points were captured by the soil sensor to assess *I_SD_*. Nearly one half of the experimental field had a lower crop leaf cover than 14%, which refers one leaf crop growth stage. The other half was also nearly at one- or two leaves stage. Weed density was more abundant in areas where the crop was less dense, *i.e.*, crop leaf cover ≤ 12%. The dominant weed species in experiments A and B were: Persian speedwell (*V. persica*), common chickweed (*S. media*), and Red Deadnettle (*L. purpureum*), accounting for 70% of the weed infestation, and European field pansy (*V. arvensis*), Scentless mayweed (*M. inodora*) and other species accounting for 30%. The weed density ranged from about 40 to more than 100 plants m^-2^. Soil density was heterogeneously distributed throughout the field and showed no clear spatial patterns; it varied from 20 to nearly 100 N at each 6 m × 6 m subplot. This information was averaged per subplot and introduced into the LFIS to derive the intensity levels and formulate the application map. The free geographical information system OpenJUMP Pirol Edition, GNU [31], was used to create the application map (Figure 4(d)). The variable harrowing intensities were applied off-line with the prototype for automatic adjustment of the harrow (Figure 1).

Two timings of harrowing, BBCH 24 and 28, were used for both experiments, since the soil crust after the winter reduced workability and not enough soil cover was produced to control weeds effectively. At both timings the harrowing treatments were the same, except at BBCH 24 one pass with the harrow was used at a driving speed of 8 kmh^−1^, and at BBCH 28 two passes at 10 kmh^−1^. Bispectral cameras and manual counting were used to assess crop leaf cover and weed density reduction immediately after harrowing, in a similar frequency as in Section 2.3. At crop maturity, yield was assessed with an automatic yield mapping system that uses a gravimetric measuring device mounted on the combine harvester (New Holland Agriculture). Statistical data analysis was done with PROC MIXED in SAS (SAS version 9.1, SAS Institute Inc, NU Cary, 2004). Unlike standard analysis of variance, mixed models include random effects (variance components) as part of the error term. The F-test allowed to compare the variances of the fixed effects of the intensity treatments, excluding the variances of the random effects of the rolling treatments nested within intensity levels and replicate blocks. The F-tests were applied with a significance level of 0.05 to test fixed effects on weed density reduction and yield. An exponential spatial covariance structure was accounted for the subplots, nested within the rolled strips with the function “type = sp(exp)”. The Tukey's Studentized Range Honest Significant Difference test (Tukey HSD) was applied to identify differences among harrowing intensities and rolling passes, regarding weed density reduction.

## 3. Results and Discussion

### 3.1. Fuzzy Inference System and Experimental Application

In general, for both experiments, weed density was effectively reduced by harrowing. In experiment A, varying the intensity according to the measured variability of soil and weeds increased the weed reduction (*P* = 0.09). F-test in the mixed model and Tukey (HSD) ranking showed that weed density was reduced in comparison with the untreated plots in experiments A (*P* < 0.001) and B (*P* < 0.0001), Table 4. In experiment A, contrasts between the varied intensity throughout the whole plot against the individual fixed intensities suggested that a higher weed density reduction might be achieved (*P* = 0.009) when the intensity is not kept constant along the cultivated plot. Similarly, Søgaard [5] found that changing the tine angle of the harrow along the whole plot reduced the variations in working depth, thus soil cover and weed control would be uniform. In experiment B, although varying the harrowing intensity effectively reduced weed density compared with the untreated control (*P* < 0.001), there was no difference in the way of varying the intensity, *i.e.*, according to either soil- or weed occurrence or a combination of both assessments (*P* = 0.42). Soil density was not influenced by the 1 to 3 passes with the roller, thus the desired high, medium and low levels of compaction could not be achieved. Therefore, soil density assessed before harrowing was almost constant across the whole experimental area (Figure 4(c)).

In theory, increasing harrowing intensities results in higher weed control at the risk of raising crop damage due to soil cover [9,12]. The crop harrowed at both BBCH 24 and 28 showed a good anchorage to the soil, hence a higher resistance to being covered by soil. Generally, crop plants will be more resistant to being covered by soil if harrowing is done at growth stages close to stem elongation [32]. Nevertheless, in experiment A crop leaf cover tended to be reduced by the strongest harrowing intensities (*P* = 0.05), while the variable intensity did not significantly affect the crop (*P* = 0.59). Consequently, the crop damage due to soil covering the crop as a result of harrowing was diminished after adjusting the intensity to the variable field conditions, as also found for winter wheat by Engelke [6].

The aim of site-specific harrowing is to avoid yield losses and to secure weed density reduction at the whole field level. Reducing unnecessary passes with the harrow or applying strong intensities on areas with high weed and/or soil densities might help to accomplish this aim. The variable intensity treatment in experiment B tended to reduce crop leaf cover (*P* = 0.07), but slightly improving crop yield (*P* = 0.13), as seen in Table 4. This increment in crop yield was not significant, thus we could not calculate an optimal intensity in any of the two experiments. It seemed that weed competition was very low, because the untreated plots showed similar crop yields as the harrowed ones. This fact confirmed the assumption that within the two week lag between assessments and harrowing, there was no significant weed development.

Furthermore, harrowing with a constant intensity along the main plot (80m) required continuous operation of the vehicle, hence a higher use of fuel. In experiment B, varying the intensity reduced the treated area, thus offering fuel saving possibilities. According to ÖKL [33], about 3.5 L ha^−1^ of fuel are needed for harrowing. In Figure 4(d), it can be seen that at least a 50% of the treated plots did not require harrowing. The highest potential to reduce fuel consumption was when the harrowing intensity was given by soil and weed variation simultaneously. Harrowing based on weed density only or soil density only determined more area to be treated. However, the test experiments revealed that weed density was the most influential input for decision making about the harrowing intensity, because the soil density was relatively homogeneous across the field.

### 3.2. Reliability of the Fuzzy Inference System for the Automatic Control of Intensity

The relationships between crop soil cover (%) and weed control (%) and the yield response studied during our previous experimental period (2007 to 2009) were the reference to delimit our input and output variables for the decision system. Our most relevant findings were that leaf cover ranged from 2% to 20%, being lower at early crop growth stages. Weed densities varied across experimental fields from 40 to about 250 plants m^-2^ and soil density varied from 9 to 19 N in spring cereals and from 23 N (autumn) to 153 N (end of winter) in winter cereals. Generally for winter cereals, harrowing in autumn generates high degrees of crop soil cover because the soil is still loose, but in early spring the soil may become highly compact, forming a crust that increases the soil density [34]. The intensity levels taken from the 2007–2009 experiments (*cf.* Table 2) attained 80% weed control with a range of 16%–30% crop soil cover. In cases of high weed competition and denser soils, nearly 45% crop soil cover was necessary to achieve 80% weed control [16]. In contrast, other studies suggested a maximum of about 25% crop soil cover [13]. However, higher degrees of crop soil cover than 25% resulted in 3% to 45% yield gain in case of high weed competition, and in cases of poor weed competition the yield loss effect due to harrowing was lower than 1% [16].

Previous attempts to adjust the harrow automatically have shown that it is not easy to define a standard intensity for every field, because it depends on the crop growth stage, weed infestation and soil conditions [6]. In a study by Søgaard [5], variations in the working depth were considered as the deciding variable to characterize the harrowing intensity, but crop growth stage and weed abundance were not considered in that system. Engelke [6] broadened the approach by including soil density, soil structure, crop- and weed growth stage at the time of harrowing and site-specific weed distribution. However, neither selectivity nor crop resistance and recovery to crop soil cover were acknowledged in both studies. Our proposed harrowing system is advantageous owing to two main reasons. Firstly, it integrates relationships between crop soil cover, weed control and yield increase as a result of harrowing, e.g., as suggested by Weis *et al.* [1], and Rasmussen *et al.* [13]. Secondly, the variables required to determine those relationships (*i.e.*, crop leaf cover, weed density and soil density) were objectively assessed and accurately analyzed with robust scientific methods [11,12,16].

The presented system does not include single weed species as input for the inference procedure. For the time being, this issue may be unimportant because broadcast cultivation with the harrow affects both the crop and the weeds, and single weed species recognition still requires further development. However, adjustment of the harrowing intensity for site-specific weeding may be also adapted to single weed species in the future. In this study, sowing depth and pre-emergence harrowing have not been investigated. Nevertheless, soil density and weed density (*i.e.*, when weeds emerge earlier than the crop) could be used as inputs to the LFIS to adjust the harrowing intensity in future experiments including pre-emergence treatments. Further validation of the LFIS is required, and experiments should include fields with variable soil types and competitive weed infestations, to illustrate the weed control effect when varying the intensity. Differing soil textures would be more accurate to improve the fuzzy decision making based method for site-specific harrowing, rather than artificially creating variable compaction levels with the roller. Additionally, weather conditions after harrowing must be investigated as well. The created rules may be the starting point to reorganize a new decision method, provided improved sensor assessments of variable conditions among different fields. A future perspective is that a real-time intensity adjustment should be achievable. The system could include attached cameras in the front and at the rear or sides of the harrow. Then, additional feedback information about the remaining weed competition on the harrowed area might be a new input to the model that would indicate the necessity of cultivating a second or more passes.

## 4. Conclusions

Valuable information was acquired in previous experiments and combined in this study with expert knowledge to formulate simple rules and develop a system to automatically control the harrow intensity. The fuzzy inference system (LFIS) was fairly well adapted to the variability of crop-weed-soil conditions in the field. Application of the LFIS for automatic harrowing in this study did not reduce crop yield under low weed competition. Further experiments under high weed density scenarios would reinforce the weed control potential of the system avoiding yield losses.

## Figures and Tables

**Table 1.** Experimental site and treatment description for the trials conducted during 2007–2009, to analyse selectivity and yield response to harrowing. Different tine angles, driving speeds and number of passes constituted the harrowing intensities, which were applied all at differing crop growth stages. Experiments were placed at various location with differing soil types and weed populations.

**Experiment (Year)**	**Crop (BBCH-Code)**	**Location (Soil Type)**	**Dominant Weeds**	**Harrowing Intensity**			**Source**
				**Tine Angle**	**Speed (km h**^−1^)	**Passes**	
(2007)							
1	winter barley	Heidfeldhof	*Lamium purpureum* L.,	lightest	10	1	Rueda-Ayala and Gerhards [15]
	(12, 24)	(silty loam)	*Galium aparine* L.,	light	10	1	
			*Alopecurus myosuroides* Huds.,	strong	12	2	
			*Matricaria inodora* L.	strongest	12	2	

(2008)							
2	spring barley	Meiereihof	*Lamium purpureum* L.,	lightest	8	1–3	Rueda-Ayala and Gerhards [15]
	(13, 21, 24)	(silty loam)	*Polygonum convolvulus* L.,	light,	8	1–3	
			*Amaranthus retroflexus* L.,	strong	12	1–3	
			*Chenopodium album* L.				
	
3	winter wheat	Heidfeldhof	*Matricaria inodora* L.,	strongest	12	1–4	Rueda-Ayala *et al.* [16]
	(12, 15, 21)	(silty loam)	*Cirsium arvense* (L.) Scop.,				
			*Alopecurus myosuroides* Huds.,				
			*Galium aparine* L.				
	
4	winter wheat	Ihinger Hof	nwc†	light	8	1–4	Rueda-Ayala *et al.* [16]
	(20)	(loam)					
	(22, 24)				10	1–4	

(2009)							
5	spring barley	Heidfeldhof	*Lamium purpureum* L.,	lightest	8	1	Meiser [18]
	(14)	(silty loam)	*Galium aparine* L.,	light	8	1	
			*Cirsium arvense* (L.) Scop.,	strong	8	2	
			*Avena fatua* L.	strongest	8	2	
	
6	spring wheat	Meiereihof	*Chenopodium album* L.,	light	8	1–3	Meiser [18]
	(12, 15)	(sandy loam)	*Veronica hederifolia* L.,				
			*Galium aparine* L.,	strong	10	1–3	
			*Alopecurus myosuroides* Huds.,				
			*Avena fatua* L.				

† nwc: no weed competition due to absence of weed emergence.

**Table 2.** Optimal intensities for the trials conducted during 2007–2009 (*cf. *Table 1) and their effects on selectivity (calculated crop soil cover corresponding to 80% weed control) and yield response (calculated crop soil cover and weed control that attained yield gain). Ranges of crop leaf cover, weed density and soil density in the untreated plots were used as data source to develop the decision making based method for automatic harrowing.

**Crop growth stage** (**BBCH**)	**Crop leaf cover** (**%**)	**Weed density** (**plants m^−2^**)	**Soil density** (**Newton**)	**Intensity**†	**Crop Soil Cover** (**%**) **corresponding to 80%weed control**	**Crop Soil Cover** (**%**) **that attained yield gain**	**Weed control** (**%**) **that attained yield gain**	**Yield gain** (**%**)
Experiment 1								
(12)	*nd*	40–56	*nd*	strong		24	94	19
(14)	18–20	82–93	*nd*	strong	47–50	15	41	16

Experiment 2								
(24)	24–27	31–41	*nd*	lightest	7–16	11	80	−3

Experiment 3								
(15)	3–5	58–73	107–147	strongest	22–30	36	90	37
(21)	8–11	148–250	101–142	strongest	22–30	28	91	45

Experiment 4								
(24)	5–9		23–25	light		16–31		0

Experiment 5								
(14)	4–6	28–63	9–20	strong	40–50	*nd*	*nd*	*nd*

Experiment 6								
(12)	6–7	58–147	*nd*	light	22–30	25	90	16
(15)	17–19	147–154	*nd*	strong	40–48	50	80	5

† Tine angles: lightest = 61°, light = 41°, strong = 28°, strongest = 4°;

*nd* = data could not be assessed.

**Table 3.** Fuzzy rule-base to infer the harrowing (*O_HI_*) (none, lightest, light, strong, strongest) for site–specific harrowing, after three levels (low, medium, high) of the variables crop leaf cover (*I_LC_*) and soil density (ISD), and four levels (none, low, medium, high) of the variable weed density (*I_WD_*).

**Input variables**							**Output variable**

**IF**	***I****_LC_*	**AND**	***I****_SD_*	**AND**	***I****_WD_*	**THEN**	**O_HI_**
	low		low		none		none
	medium		low		none		
	high		low		none		
	low		medium		none		

	medium		medium		none		lightest
	high		medium		none		
	low		high		none		
	medium		high		none		
	high		high		none		
	low		low		low		
	medium		low		low		
	high		low		low		
	low		medium		low		
	medium		medium		low		
	low		medium		medium		

	high		medium		low		light
	low		high		low		
	medium		high		low		
	high		high		low		
	low		low		medium		
	medium		low		medium		
	high		low		medium		
	medium		medium		medium		
	low		high		medium		
	medium		high		medium		
	low		low		high		
	medium		low		high		
	low		medium		high		
	low		high		high		

	high		medium		medium		strong
	high		high		medium		
	high		low		high		
	medium		medium		high		
	medium		high		high		

	high		medium		high		strongest
	high		high		high		

**Table 4.** Effects of harrowing treatments on leaf cover, weed density and crop yield in experiments A and B.

**Responses after harrowing**				
**Harrowing treatment**	**rolling** (**passes**)	**Leaf cover**^ns^ (**%**)	**Weed density**† (**plants m^−2^**)	**Crop yield**^ns^ (**t ha^−1^**)
Experiment A				
none	1	28.7	18.0 *b*	5.9
	2	27.7	27.2 *b*	6.4
	3	25.9	22.3 *b*	5.9
light	1	23.2	14.3 *a*	6.4
	2	21.9	12.7 *a*	6.3
	3	20.7	8.7 *a*	6.6
strong	1	22.7	11.8 *a*	6.8
	2	23.5	13.9 *a*	6.1
	3	22.5	8.1 *a*	6.4
strongest	1	21.3	7.1 *a*	6.5
	2	22.1	9.0 *a*	6.4
	3	22.6	8.8 *a*	6.8
varied	1	24.2	9.5 *a*	6.6
	2	23.0	7.8 *a*	6.7
	3	24.5	8.4 *a*	6.7

Experiment B				
untreated	1	30.8	26 *B*	5.7
	2	28.0	28.5 *B*	6.7
	3	26.0	23.1 *B*	6.2
*I_SD_*	1	23.0	8.1 *A*	6.6
	2	24.3	8.4 *A*	6.8
	3	23.4	4.1 *A*	7.0
*I_WD_*	1	23.6	4.2 *A*	6.3
	2	25.4	6.3 *A*	6.9
	3	23.9	5.2 *A*	6.8
*I_SD_* + *I_WD_*	1	24.3	7.8 *A*	6.9
	2	23.5	6.1 *A*	6.8
	3	23.3	4.7 *A*	6.4

ns non-significant effects;

† Tukey (HSD) ranking at α = 0.05, small letters for experiment A and capital letters for experiment B

## References

[1] Weis M., Gutjahr C., Rueda-Ayala V., Gerhards R., Ritter C., Schölderle F. (2008). Precision farming for weed management: Techniques. Gesunde Pflanzen.

[2] Slaughter D.C., Giles D.K., Downey D. (2008). Autonomous robotic weed control systems: A review. Comput. Electron. Agric..

[3] Gerhards R., Oebel H. (2006). Practical experiences with a system for site-specific weed control in arable crops using real-time image analysis and GPS-controlled patch spraying. Weed Res..

[4] Griepentrog H.W., Nørremark M., Nielsen J. Autonomous Intra-row Rotor Weeding Based on GPS.

[5] Søgaard H.T. (1998). Automatic control of a finger weeder with respect to the harrowing intensity at varying soil structures. J. Agric. Eng. Res..

[6] Engelke B. (2001). Entwicklung eines Steuersystems in der ganzflächig mechanischen Unkrautbekämpfung [Development of a self-adjusting system for broadcast mechanical weed control]..

[7] Kurstjens D.A.G., Kropff M.J. (2001). The impact of uprooting and soil-covering on the effectiveness of weed harrowing. Weed Res..

[8] Spoor G., Godwin R. (1978). An experimental investigation into the deep loosening of soil by rigid tines. J. Agric. Eng. Res..

[9] Cirujeda A., Melander B., Rasmussen K., Rasmussen I.A. (2003). Relationship between speed, soil movement into the cereal row and intra-row weed control efficacy by weed harrowing. Weed Res..

[10] Rasmussen J., Nørremark M., Bibby B.M. (2007). Assessment of leaf cover and crop soil cover in weed harrowing research using digital images. Weed Res..

[11] Rasmussen J., Bibby B.M., Schou A.P. (2008). Investigating the selectivity of weed harrowing with new methods. Weed Res..

[12] Rasmussen J., Nielsen H.H., Gundersen H. (2009). Tolerance and selectivity of cereal species and cultivars to postemergence weed harrowing. Weed Sci..

[13] Rasmussen J., Mathiasen H., Bibby B.M. (2010). Timing of post-emergence weed harrowing. Weed Res..

[14] Rasmussen J. Selectivity-an Important Parameter on Establishing the Optimum Harrowing Technique for Weed Control in Growing Cereals.

[15] Rueda-Ayala V.P., Gerhards R., van Henten E., Goense D., Lokhorst C. (2009). Selectivity of Weed Harrowing with Sensor Technology in Cereals in Germany.

[16] Rueda-Ayala V.P., Rasmussen J., Gerhards R., Fournaise N.E. (2011). The influence of post-emergence weed harrowing on selectivity, crop recovery and crop yield in different growth stages of winter wheat. Weed Res..

[17] Zadeh L. (1965). Fuzzy sets. Inf. Control.

[18] Meiser B. (2009). Selektivität in de Mechanischen Unkrautbekämpfung mit einem Striegel in Getreide [Selectivity in Mechanical Weed Control with A Flexible-tine Harrow in Cereals]. M.Sc. Thesis.

[19] Rueda-Ayala V.P., Rasmussen J., Gerhards R., Oerke E.C., Gerhards R., Menz G., Sikora R.A. (2010). Mechanical Weed Control. Precision Crop Protection–the Challenge and Use of Heterogeneity.

[20] Xia F., Zhao W., Sun Y., Tian Y.C. (2007). Fuzzy logic control based QoS management in wireless sensor/actuator networks. Sensors.

[21] Valente de Oliveira J. (1995). A design methodology for fuzzy system interfaces. IEEE Trans. Fuzzy Syst..

[22] Yang C.C., Prasher S.O., Landry J.A., Ramaswamy H.S. (2003). Development of a herbicide application map using artificial neural networks and fuzzy logic. Agric. Syst..

[23] Sivanandam S., Sumathi S., Deepa S. (2007). Introduction to Fuzzy Logic Using MATLAB.

[24] Wong S.V., Hamouda A.M.S. (2003). The development of an online knowledge-based expert system for machinability data selection. Knowl. Based Syst..

[25] Bosma R., Kaymak U., van den Berg J., Udo H., Verreth J. (2010). Using fuzzy logic modelling to simulate farmers'decision-making on diversification and integration in the Mekong Delta,Vietnam. Soft Comput. A Fusion Found. Methodol. Appl..

[26] Klose A., Nuernberger A. Applying Boolean Transformations to Fuzzy Rule Bases.

[27] Zhou S.M., Gan J.Q. (2008). Low-level interpretability and high-level interpretability: A unified view of data-driven interpretable fuzzy system modelling. Fuzzy Sets Syst..

[28] Marakoglu T., Carman K. (2010). Fuzzy knowledge-based model for prediction of soil loosening and draft efficiency in tillage. J. Terramechanics.

[29] Nurcahyo G., Shamsuddin S., Alias R. (2003). Selection of defuzzification method to obtain crisp value for representing uncertain data in a modified sweep algorithm. J. Comput. Sci. Technol..

[30] Steinmann H.H. (2002). Impact of harrowing on the nitrogen dynamics of plants and soil. Soil Tillage Res.

[31] FSF (1999). GNU General Public License..

[32] Kurstjens D.A.G., Perdok U.D. (2000). The selective soil covering mechanism of weed harrows on sandy soil. Soil Tillage Res..

[33] ÖKL Kraftstoffverbrauch in der Land-und Forstwirtschaft [Fuel usage in agriculture and forestry]..

[34] Rasmussen J., Nørremark M. (2006). Digital image analysis offers new possibilities in weed harrowing research. Zemdirbyrste.

